# Leisure Hours in Physic and Surgery

**Published:** 1827-07

**Authors:** 


### LEISURE HOURS IN PHYSIC AND SURGERY.

1. *The Gold-headed Cane.* 8vo, pp. 180. 1827.
2. *Nugæ Canoræ; or Epitaphian Mementos (in stone-cutters' verse) of the Medici Family, of Modern Times.* By UNUS QUORUM. 8vo, pp. 70. Callow and Wilson, 1827.

WHEN we read the title page of the first work on our list, we thought we had in our hands a novel. It is a medical novel—at least it is a novelty in medicine. It is evidently an imitation of the GREAT UNKNOWN. And had this LITTLE unknown exercised his talent (which is by no means inconsiderable) in sketching the manners of medical society—the state of medical doctrine and practice—the life and characters of those medical men who figured on the stage during the long life and change of masters which the gold-headed cane experienced, he might have made his little work extremely interesting to his professional brethren. But, instead of this plan, the author has not dedicated one tenth of the 180 pages to purely medical biography. He has occupied his pen in sketching the characters or relating anecdotes of Kings, Princes, Queens, and Princesses, Courtiers, Ministers, &c. by which proceeding, the work is rendered a perfect NON-DESCRIPT.

The Gold-headed Cane is no imaginary being; though the author has thought proper to endow this said GOLD-HEAD with brains, eyes, ears, and intellects—nay, with a tongue to speak, and a hand to write certain things that must prove a most flattering unction to the souls of *some people.** This cane was the slave of Radcliffe, Mead, Askew, Pitcairn, and Baillie—by the latter of whom *manumission* was granted to this faithful bondsman and servant. In one corner of Dr. Baillie's consultation-room, formerly, and now in a closet of the new college, the Gold-headed Cane has been allowed to put on his PILEUS, or cap of liberty, in token of his release from all future service. It was in this last retreat that he bethought himself of writing a memoir of his own life, and of the masters through whose hands he had passed.

It is a wise ordination of Providence, that intellectual property is not transmissible or bequeathable from one generation to another, like goods and chattels—in other words, that the *inside* furniture of the head cannot be *willed* away, like the *outside* furniture. Were it otherwise than it is, we should have the brains, as well as the wigs and canes of Mead, Heberden, Pitcairn, Baillie, &c. all carefully preserved in Pall Mall East, instead of having undergone the mysterious process of incarnation, in other shapes and forms.

This beneficent dispensation of Nature provides against monopoly of talent, and secures a diffusion of intellect, in despite of all human devices for the attainment of the one or prevention of the other.

We shall now glance at some of the medical portraits presented in this little volume, professing our total ignorance of the author who has drawn them.

### I. RADCLIFFE.

It was in the autumn of 1689, that the Gold-headed Cane first made his debut with his master, in the sick chamber of William the Third, at Kensington. Dr. Bidloo was in at-

* The motto which Dr.—— might have taken for the Gold-headed Cane, is, we think, the following:—

Tu pias lætis *animas* reponis

Sedibus; *virgaque* levem coerces

*Aurea* turbam, superis deorum

Gratus et *imis*. HOR.

tendance on his Majesty, and the scene opens with a speech from Dr. Radcliffe, not less remarkable for its trickery, than for its quaint pathology. Dr. Radcliffe pursued the identical course, upon this occasion, which all illiberal practitioners pursue at the present day—namely, by conveying insinuations against others, to magnify their own merits, and thus ingratiate themselves with the patients to whom they are called in consultation.

"May it please your Majesty," said Dr. Radcliffe, "I must be plain with you, Sir:—your case is one of *danger*, no doubt; but if you will adhere to *my* prescriptions, *I* will engage to do you good. *The rheum is dripping on your lungs*, and will be of fatal consequence to you, unless it be otherwise diverted."

A more exquisite specimen of trickery than the above, is not, we venture to aver, on record. It was no wonder that the choleric Bidloo was up in arms on hearing such an illiberal and selfish declaration from the new physician; and a battle-royal would have been the result, had not the King ordered the physicians out of his presence, to settle their disputes in another room.

There is little in Dr. Radcliffe's life which is worth recording in this place. His practice rapidly increased—that's certain: but by what means? "It was clear," says the Gold-head Cane, "his erudition had nothing to do with it; but though there was something rude in the manner in which he frequently disparaged the practice of others, yet it could not be denied that his general good sense and practical *knowledge of the world* distinguished him from all his competitors." This knowledge of the world was a poor excuse for his illiberal disparagement of his brethren, though we fear it prompted a little to the practice.

One of the most remarkable instances of *second sight*, for we cannot call it *prognosis*, is recorded at page 21, of the work before us. "Being sent for once to attend the Duke of Beaufort at Badminton, who was very ill, the Doctor, instead of complying with the request, told the gentleman who brought the message—"there was no manner of necessity for his presence, since the Duke, his master, died such an hour the day before." The messenger returned, and found the prognosis correct! This needs more confirmation than comment.

Political revolutions banished the once celebrated Doctor to a rural retirement, where he died, apparently of ennui and dread of the populace.

### II. MEAD.

From a master of sagacity and practical acquirements, the Gold-headed Cane passed into the hands of an accomplished scholar and physician—Mead, who was characterised, even by his antagonists, as "artis medicæ decus." His treatise on poisons—his resistance to the abolition of quarantine laws—and his advocacy of inoculation, are the three principal features of Mead's professional regency. It is curious, as the author before us observes, that the contagious property of small-pox was never discovered, till the new method of conveying it by inoculation was introduced from the East. The contagion of small pox totally escaped the observation of Sydenham. We were rather surprised that so great a parade should be made of one of this physician's improvements in the practice of medicine—viz. the application of pressure to the abdomen, while water was drawn off by a trocar—and a bandage subsequently. Certainly the discovery (if this were really the first time that such a step was thought of) is not of the brightest cast; but the periphrastic and almost unintelligible explanation which is given of the modus operandi of the tapping in causing faintness, is quite a morceau.

"This was certainly a *most valuable discovery*, and shewed the advantage derivable from the exercise of good sense and sound judgment: for Mead naturally reflected, *that the removal of the pressure of the accumulated water caused the fibres suddenly to lose the extension which they had previously acquired; and it as naturally occurred to him, that the tendency to faint could only be obviated by substituting an external support to the parts.*"

The whole of the above periphrase might have been expressed in three or four words:—"for the pressure of the water on the viscera and vessels is substituted the pressure of a bandage."

Passing over Askew and Pitcairn, the golden-headed memoirs of whom contain nothing worthy of remark, we must dwell a few moments on the last master of the Cane, by introducing an extract from Sir Henry Halford's eloquent observations on the life and character of his friend and contemporary.

"The attention which he had paid to morbid anatomy (that alteration of structure which parts have undergone by disease) enabled him to make a nice discrimination in symptoms, and to distinguish between disorders which resemble each other. It gave him a confidence also in propounding his opinions, which our conjectural art does not readily admit; and the reputation, which he enjoyed universally for openness and sincerity, made his *dicta* be received with a ready and unresisting faith.

"He appeared to lay great stress upon the information which he might derive from the external examination of the patient, and to be much influenced in the formation of his opinion of the nature of the complaint by this practice. He had originally adopted this habit from the peculiar turn of his early studies; and assuredly such a method, not indiscriminately but judiciously employed, as he employed it, *is a valuable auxiliary to the other ordinary means* used by a physician of obtaining the knowledge of a disease submitted to him. But it is equally true that, notwithstanding its air of mechanical precision, such an examination is not to be depended on beyond a certain point. Great disordered action may prevail in a part, without having yet produced such disorganization as may be sensibly felt; and to doubt of the existence of a disease because it is not discoverable by the touch, is not only unphilosophical, but must surely, in many instances, lead to unfounded and erroneous conclusions."

The truly enlightened and talented President is right in the above remarks. To attempt to discover disordered action by thoracic percussion, or abdominal pressure, is quite absurd. But the great value of these external investigations consists in the power of often distinguishing when organic disease has actually taken place—a power which, an examination of the mere symptoms of the malady will not always, or perhaps even generally, confer on the physician. But accurate auscultation will go beyond the other means of external investigation; and often disclose disordered *function* in the heart and lungs, before any material change of structure has taken place. It will reveal the extent, for instance, of pleuritic inflammation—inflammation of the mucous membrane of the bronchia, &c. which can hardly be called organic disease, and which cannot be so well ascertained by the symptoms alone.

But we must take our leave of the Gold-headed Cane, and its learned author; regretting, as we said before, that he did not employ his talent in medical biography and manners, rather than in sketches of character beyond the pale of the profession.

---

It is now time that we should turn to the other production at the head of our list, emanating, it is said, from the pen of a surgeon not unregistered on the rolls of Fame. The publications now before us give us hope that the glorious days of Smollet, Garth, Goldsmith, Darwin, &c. are returning; and that physicians and surgeons, of talent, will employ their *leisure* hours in novels and poetry. If they do, we predict that Pater Noster Row will not be able to disgorge, from its dark unfathomable caverns, the volumes manufactured by this class of intellectual operatives.

The work entitled "Nugæ Canoræ" was suggested by the emigration of the College of physicians from Warwick Lane to Pall Mall East. It struck the author that it would be no bad thing, in these days of speculation, to have a "MEDICAL DEATH SOCIETY," and he suggests the old College as "a MEDICAL MAUSOLEUM," or Pantheon Medicum, where—

"The self same Doctors may appear in shrouds,"

when they have sent their quota of patients to the Elysian Shades. After arranging all the minutiæ of this *Pere la Chaise*, in Warwick Lane, the author employed himself in writing epitaphs, "in stone cutters' verse," on the worthies who were to be deposited in the consecrated spot. The work is dedicated to the Presidents of the two Colleges, and to the Master of the Company of Apothecaries, for the year 1927—a liberty which he thinks he may safely take, as he is not very likely to meet these official characters, in consultation, a century hence. In introducing some of his contemporaries to these regents of 1927, he expresses his humble hope "that the honorable profession of medicine may hold the same elevated rank in the scale of society, to which its present professors have raised it—and that the Abernethys, Clines, and Coopers of one college, and the Ainslies, Halfords, and Warrens of the other, may be as distinguished ornaments of them in their day, as in his own—and that science and surgery, philosophy and physic, may continue synonymous to the remotest ages of posterity." We say, amen! But we might remark that, as a member of the College of Surgeons, it would have been in better taste, had the author of the NUGÆ CANORÆ placed the members of the *other* College first on the list, in his prospective introduction to posterity. At an army dinner, the toast is always the "navy and army"—and at a naval dinner—"the army and Navy." We think the same courtesy might well be observed upon other occasions, and especially on the present.

Passing over Heberden, Turton, Baker, De Castro Sarmento, and Sir Richard Jebb, of whom very short biographical notices are given, we come to Fordyce, whose habits of eating and drinking are commemorated. It appears that Dr. F. dined every day at Dolly's Chop-house, for more than twenty years, in the following manner:—At 4 o'clock, the little side table was always laid, with a silver tankard full of strong ale, a bottle of port wine, and a quarter of a pint of brandy.

"The moment the waiter announced him, the cook put a pound and a half of rump-steak on the gridiron, and on the table some delicate trifle as a *bonne bouche*, to serve until the steak was ready. This was sometimes half a broiled chicken, sometimes a plate of fish: when he had eaten this, he took one glass of brandy, and then proceeded to devour his steak. When he had finished his meal, he took the remainder of his brandy, having, during his dinner, drunk the tankard of ale, and afterwards the bottle of port! He thus daily spent an hour and a half of his time, and then returned to his house in Essex-street, to give his six o'clock Lecture on Chemistry. He made no other meal until his return next day, at four o'clock, to Dolly's." 8.

Dr. Fordyce, like some modern members of the Medici family, had certain peculiar opinions respecting the "digestive organs" of man. He happened to observe that the noble lion made only one meal a day—and, *hence,* he concluded that man should do the same! It must be allowed that the doctor took a lesson at Exeter Change, and, like the lion, made a—"mortal gorge," when he did set to! The epitaph however, does not convey a very pressing invitation to posterity to follow his example.

"The reason why I'm here interr'd,
Methinks may rightly be referr'd
To living *well* and drinking *hard.*
Should you, dear patients, then prefer,
Death's final visit to defer,
Shun Aqua Vitæ and *Mollard.*" 7.

---

Dr. Reynolds is characterized as the link between the ancient and modern costume of the faculty. "He wore a well powdered wig and a silk coat." These insignia are now laid aside —for ever!

The epitaph on Dr. Curry, of Guy's Hospital, contains the well-known anecdote of the mistake which the doctor made respecting his own case, considered to be a liver complaint, but which turned out to be disease of the lungs. If the number of mistakes, *of this kind,* made by some modern physicians, of no mean note, were registered, the laugh would not be so great against poor Curry as it now is.

The anecdotes of Lettsom are not new, and the epitaph is certainly in "*stone-cutters' verse.*"

One of the best epitaphs in the book is that on Dr. Lambe— who, by the bye, is not likely, for some years yet, to have a stone over his mortal part. We are tolerably quick in our pace; but the water-drinking doctor fairly beat us the other day, in walking up Highgate Hill.

Ἄριστον μὲν ὕδωρ.

"Here lies a man who, drinking only water,
Wrote several books, with each had son or daughter;
Had he but used the juice of generous vats,
The World would scarce have held his books and brats;
Or had he not in pulse been such a glutton,
This LAMB had not been now as dead as mutton." 17.

---

Drs. Moseley and Rowley are commemorated in "stone-cutters' verse"—not much to their credit. Peace to their manes! How the author could suppose that these two worthies—

" ——— purged the schools of solecism
Refin'd pedantic barbarism,"

we do not know; but this we will say, that Dr. Moseley's *best* work is a tissue of "pedantic barbarism itself."

---

The following epitaph, though now *written*, will not, we hope, be *engraved* on the tomb-stone of the worthy Dr. S——e for 30 years to come.

" Here, snug in a corner,
Like little Jack Horner,
Lies a Doctor who wrote on Bath water;
By his works you will find,
Should you be so inclin'd,
He to Death and Disease gave no quarter." 24.

---

Dr. Denman is eulogized in high terms. The following is not amiss.

" Beneath this stone, shut up in the dark,
Lies a learned man-midwife, y'clep'd Doctor Clarke.
On earth while he lived, by attending men's wives,
He increas'd population some thousands of lives:
Thus a gain to the nation was gain to himself;
And enlarg'd population enlargement of pelf.
So he toil'd late and early, from morning till night,
The squalling of children his greatest delight.
Then worn out with *labours*, he died skin and bone,
And his ladies he left all to *Mansfield* and *Stone*." 27.

The epitaph on Dr. Gall or Dr. Spurzheim (for it may do for either) is not very clever. The following one, on (we believe) Mr. Earle, is rather better.

" To shew that, unlike to old drones,
Young surgeons are full of invention,
Here lies one who did add to the bones,
A bone—called *the bone of contention*."

We suppose the neck of the thigh-bone is here alluded to, about the fractures of which so much contention recently prevailed—and in which Sir A. Cooper certainly had the best of the battle.

We can add but one more epitaph. Whether the stone on which it is to be engraved shall be seen in a *place* called Whitehall, or *close* to Bartholomew's Hospital, is yet a secret in the womb of fate.

"Here lies a warm spirit, whose genius and fire
Caused his death, from the heat of his passion and ire.
For so scorching and hot was his learning and knowledge,
It *embroiled* the profession, and *roasted* the College." 50.

---

And shall the humorous and good-natured author of epitaphs lie without one on his own stone? Forbid it the Muses! Let us try—in his own way,

*Epitaph on the Author of Nugæ Canoræ.*

Here lies beneath this marble hearse,
Th' inventor of "STONE-CUTTER VERSE:"—
He *carved* the living—*etched* the dead—
And thus he gained both fame and bread.
A surgeon bold—a poet sad—
A social soul was Mr. ——.

---

But, having committed ourselves thus, we shall be accused of partiality if we do not a similar charitable act for the GOLD-HEADED CANE.

*Epitaph on the Gold-headed Cane.*

Here, ripe in years—in wisdom mellow,
Reposeth one most learned "FELLOW;"
Who drew an intellectual feast,
From musty tomes in Pall Mall East;—
Then wrote a book, to prove his KNOWLEDGE,
And praise the fellows of the College.

